# 

**Published:** 1886-04

**Authors:** 


					﻿yo\. vii.
APRIL, 1886
NO.4
THE
IntatndentPraetiliontr:
> lONTHLT JOUIWL
DEVOTED TO
DENTAL AND ORAL SCIENCE.
W. C. BARRETT, M. D„ D. D. S.
EDITOR.
The Ke w York Dental Journal Association,
PUBLISHERS,
JSTo. 33 West <L6th Street, New York.
AND
sNo. £208 Franklin St., Buffalo, N.Y.
$2.50 Per Annum in Advance, .... Single Copies, 25 Cents.
Entered at the Post-Office, Buffalo, N. Y., as Second-Class matter.
				

